# Advancing cervical cancer treatment: integrating cannabinoids, combination therapies and nanotechnology

**DOI:** 10.1007/s00432-025-06323-6

**Published:** 2025-10-16

**Authors:** S. P. Mathibela, K. N. Ncube, M. T. Lebelo, V. Steenkamp

**Affiliations:** 1 Department of Physiology, University of Pretoria, Private Bag X323, Gezina, Pretoria, 0031 South Africa; 2 Department of Pharmacology, University of Pretoria, Private Bag X323, Gezina, Pretoria, 0031 South Africa

**Keywords:** Cervical cancer, Cannabinoids, Combination therapies, Drug delivery systems, Nanotechnology

## Abstract

**Background:**

Cervical cancer remains a major global health challenge, with the highest incidence and mortality rates observed in sub-Saharan Africa. Despite progress in prevention and treatment, the management of advanced and recurrent disease remains difficult.

**Aim:**

This review explores the potential role of cannabinoids in cervical cancer therapy, with a focus on their integration into existing treatment strategies, combination therapies, and nanotechnology-based delivery systems.

**Methods:**

A critical synthesis of preclinical studies and emerging therapeutic approaches was conducted, examining the anticancer properties of cannabinoids, their mechanisms of action, and their application within combination and nanotechnology-based treatment modalities.

**Results:**

Cannabinoids such as tetrahydrocannabinol (THC) and cannabidiol (CBD) demonstrate anticancer effects by inducing apoptosis, inhibiting cell proliferation, and suppressing metastasis. Mechanistic studies highlight their ability to promote oxidative stress, modulate key signalling pathways, and influence immune responses in cervical cancer cells. Combination therapies involving cannabinoids with chemotherapy, radiotherapy, and immunotherapy show enhanced efficacy and reduced drug resistance. Furthermore, nanotechnology-based delivery systems offer advantages including targeted drug release, improved solubility, controlled dosing, and decreased systemic toxicity.

**Conclusion:**

Cannabinoids represent a promising adjunct in cervical cancer management. However, successful clinical translation requires optimisation of formulations, establishment of dosing protocols, and comprehensive safety evaluation. Future research should also explore biomarker-driven personalised medicine approaches. Standardisation, along with addressing regulatory and ethical challenges, will be crucial for the integration of cannabinoid-based therapies into mainstream cervical cancer treatment.

## Introduction

Cervical cancer remains a significant global health challenge, ranking eighth among all cancers and fourth among cancers in women globally (Bray et al. 2024). In 2022, there were 661,021 new cervical cancer cases and 348,189 deaths reported worldwide, with sub-Saharan Africa (SSA) bearing the highest burden (Bray et al. 2024). Despite being preventable and treatable when detected early, many cases in SSA are identified at advanced stages, leading to poor outcomes (Burt et al. 2021). This issue is exacerbated by limited healthcare access, insufficient screening programs, and a high human papillomavirus (HPV) prevalence, which is associated with 99% of cervical cancer cases (Burt et al. 2021). Despite significant reductions in cervical cancer incidence and mortality rates due to HPV vaccination and early screening programs, challenges persist in managing advanced and recurrent cases (Burt et al. 2021). While standard treatment regimens such as surgery, chemotherapy, and radiotherapy have improved patient outcomes, they are often associated with significant toxicities, resistance, and recurrence (Burmeister et al. 2022). Consequently, there is a pressing need to explore novel therapeutic strategies that can enhance treatment efficacy while minimizing adverse effects.

In recent years, cannabinoids have emerged as promising candidates in cancer therapy due to their ability to modulate multiple cellular pathways involved in tumour progression (Daris et al. 2019). Derived from the *Cannabis sativa* plant, cannabinoids like tetrahydrocannabinol (THC) and cannabidiol (CBD) interact with the endocannabinoid system (ECS) and exhibit anticancer properties, including the induction of apoptosis, inhibition of proliferation, and suppression of metastasis (Daris et al. 2019). Preclinical studies have demonstrated that cannabinoids can exert cytotoxic effects on cervical cancer cells through mechanisms such as oxidative stress induction, modulation of key signalling pathways such as; phosphoinositide 3-kinase/protein kinase B/mechanistic target of rapamycin (PI3K/AKT/mTOR) pathway, mitogen-activated protein kinase (MAPK)/extracellular signal-regulated kinase (ERK) pathway and caspase pathway, and alteration of immune responses (Pagano et al. 2021). Despite these promising findings, challenges related to the bioavailability, pharmacokinetics, and legal status of cannabinoids hinder their clinical application in cancer therapy (Grotenhermen 2003). To address these challenges and explore potential solutions, researchers have been investigating innovative approaches to enhance the efficacy of cannabinoids in cancer treatment.

Combination therapy approaches involving cannabinoids alongside conventional treatments such as chemotherapy, radiotherapy, and immunotherapy show notable promise in cervical cancer treatment (Mokoena et al. 2024). It has been shown that cannabinoids may promote synergy when combined with chemotherapeutic agents like cisplatin, potentially enhancing their cytotoxic effects while reducing drug resistance (Chen et al. 2024). To address the limitations of cannabinoids in cancer therapy, nanotechnology-based drug delivery systems have emerged as a promising strategy to enhance their therapeutic efficacy in cervical cancer treatment (Song et al. 2024). Nanoparticle formulations offer several advantages, including targeted drug delivery, controlled release, improved solubility, and reduced systemic toxicity (Zhuo et al. 2024). Researchers have investigated various nanocarriers, such as liposomes, polymeric nanoparticles, and lipid-based nanoparticles, for encapsulating cannabinoids to enhance their stability and bioavailability (Zhuo et al. 2024). This review outlines existing treatment strategies for cervical cancer and explores novel therapeutic developments in the field. It discusses the potential role of cannabinoids in cancer management and underscores the promise of combining them with conventional therapies. In addition, it examines the contribution of nanotechnology in improving the targeted delivery of cannabinoids for cervical cancer treatment.

## Current cervical cancer treatment options

Treatment options for cervical cancer are guided by the stage and extent of cancer progression, typically including surgery, radiation therapy, and chemotherapy, either individually or in combination (Burmeister et al. 2022). While these conventional treatments have improved patient outcomes, they also have limitations such as side effects and potential inefficacies (Burmeister et al. 2022).

### Surgery

Surgery is a commonly employed and successful method for treating early-stage cancers, involving the removal of both cancerous and metastatic tissue (Burmeister et al. 2022). The type of surgery depends on the stage of the disease and its extent of spread. In cervical cancer cases, a total hysterectomy, with or without salpingo-oophorectomy (removal of one or both ovaries and fallopian tubes), is typically recommended for women who are past childbearing age (Burmeister et al. 2022). For women of childbearing age with early-stage cancer, a more conservative approach may be necessary, utilizing fertility-sparing surgeries such as loop electrosurgical excision procedure, conization, and trachelectomy (Terzic et al. 2023). While surgery has proven effective in treating early-stage cervical cancer, it has limitations and may not be sufficient for advanced stages, which often require additional therapies like chemotherapy and radiotherapy (Guimaraes et al. 2022). Surgical practice, the adoption of robotic-assisted techniques can improve the precision of tumour excision and shorten patient recovery time (Reddy et al. 2023). Advanced intraoperative imaging methods, such as fluorescence-guided surgery, enhance tumour visibility, while sentinel lymph node mapping helps limit the extent of lymph node dissection, thereby reducing the risk of related complications (Bortot et al. 2023).

### Radiotherapy

Radiotherapy, which utilizes high-energy X-rays, is also commonly used in cervical cancer treatment (Burmeister et al. 2022). The main types of radiotherapy used in treating this cancer are external beam radiotherapy, intensity-modulated radiotherapy, and brachytherapy (Burmeister et al. 2022). Despite advancements in radiotherapy techniques, several potential side effects remain, including diarrhoea, abdominal cramps, pelvic pain, skin irritation, lymphedema, and sexual dysfunction (Burmeister et al. 2022). In certain clinical settings, a complete response is observed in 68.3% of patients with early-stage cervical cancer, however, radiotherapy alone has been insufficient in reducing the spread of locally advanced disease in 20–50% of treated patients (Burmeister et al. 2022). To enhance treatment effectiveness, particularly in cases where cervical cancer lesions exceed 4 cm in width, radiotherapy is often combined with chemotherapy (Burmeister et al. 2022).

In SSA, two- and three-dimensional conformal radiotherapy remain the most commonly used techniques due to ongoing resource limitations (Diwanji et al. 2017; Hope-Johnson et al. 2025). Despite these challenges, a retrospective study in Botswana by Grover et al. (2022) demonstrated that combining radiotherapy with chemotherapy significantly improved outcomes for patients with locally advanced cervical cancer, achieving a three-year overall survival rate of 58% (Grover et al. 2022). The adoption of intensity-modulated radiation therapy (IMRT) and image-guided radiation therapy (IGRT) could enhance treatment precision by better accounting for organ motion. Additionally, investigating hypofractionated radiotherapy regimens may help shorten treatment duration and increase patient adherence.

### Chemotherapy

Chemotherapy is a cornerstone of the standard treatment plan for cervical cancer, often administered after surgery to reduce the risk of recurrence when high-risk tumour features are present (Burmeister et al. 2022). In more advanced cases, chemotherapy is typically combined with radiotherapy to enhance treatment effectiveness (Burmeister et al. 2022). Cisplatin remains the primary chemotherapy drug for cervical cancer treatment, especially in SSA, where single-agent cisplatin is commonly used due to cost constraints and limited access to newer drugs (Burmeister et al. 2022). However, research has shown that combining cisplatin with other chemotherapeutic agents can improve patient outcomes (Tewari et al. 2014). The addition of bevacizumab to cisplatin has demonstrated improved overall survival in women with recurrent, persistent or metastatic cervical cancer (Tewari et al. 2014). Combining cisplatin with topotecan increased response rates in advanced cervical cancer, while paclitaxel as a second-line therapy with cisplatin showed promising results in advanced or recurrent cases (Moon et al. 2018). Building on these advancements in combination therapies, researchers have begun exploring novel therapeutics approaches to further improve outcomes for patients with cervical cancer.

## Emerging therapeutic approaches for cervical cancer

### Cannabinoids in cervical cancer therapy

**Fig. 1:**
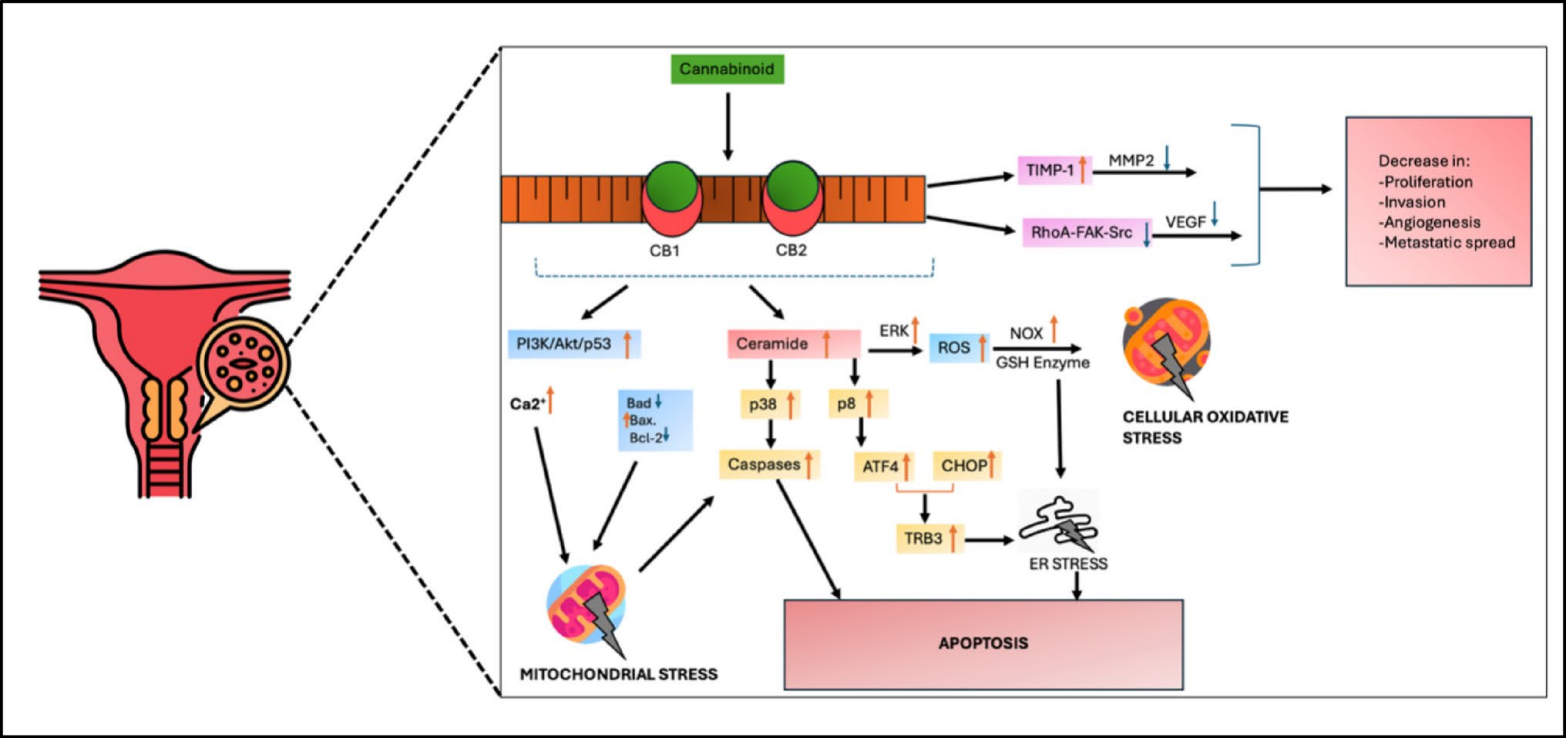
Schematic representation of endocannabinoid system (ECS) regulation and associated cell signalling pathways involved in cannabinoid-mediated cervical cancer cell death. Figure created by SP Mathibela using FlatIcon.com and PowerPoint Presentation 2021. The diagram includes various components; Cannabinoid receptors 1 and 2 (CB1 and CB2), tissue inhibitor of metalloproteinase-1 (TIMP-1), matrix metalloproteinase 2 (MMP2), reactive oxygen species (ROS), activating transcription factor 4 (ATF-4), C/EBP homologous protein (CHOP), phosphoinositide 3-kinase (PI3K), protein kinase B (AKT), B-cell lymphoma 2 (Bcl-2), Bcl-2 associated X protein (Bax), Bcl-2 associated agonist of cell death (Bad), NADPH oxidase (NOX), vascular endothelial growth factor (VEGF), calcium ion (Ca.^2+^), tribbles homolog 3 (TRIB3), Ras homologue family member A (RhoA), focal adhesion kinase (FAK) and proto-oncogene tyrosine-protein kinase (Src), glutathione (GSH)

Cannabinoids have shown promising potential in cervical cancer treatment through various molecular mechanisms involving the ECS (Daris et al. 2019). Two prominent cannabinoids, THC and CBD exhibit anti-invasive properties on cervical cancer cells, primarily through the induction of tissue inhibitor of metalloproteinase-1 (TIMP-1) expression, which is mediated through cannabinoid receptors (CB1 and CB2) (Ramer and Hinz 2008). THC and CBD upregulate TIMP-1, which inhibits matrix metalloproteinase 2 (MMP2) and blocks tumour angiogenesis and invasion (Fig. 1) (Bakshi et al. 2022). The cannabinoids induce apoptosis in cervical cancer cells by increasing the production of reactive oxygen species (ROS), disrupting mitochondrial membrane potential, activating caspase-dependent cell death pathways, upregulating pro-apoptotic proteins, and downregulating anti-apoptotic proteins (Fig. 1) (Hosami et al. 2021). In particular, CBD has demonstrated several specific anticancer properties, including inducing immune responses that obstruct tumour invasion and angiogenesis, activating the p38/MAPK pathway, modulating the RhoA-FAK-Src axis, increasing ceramide synthesis through CB receptor activation and upregulating normal p53 expression (Fig. 1) (Mashabela and Kappo 2024). These mechanisms collectively promote apoptosis and inhibit cervical cancer growth, survival, and angiogenesis (Mashabela and Kappo 2024). While these findings are promising, it is important to note that they are primarily based on preclinical and in vitro studies. Further research, especially involving in vivo models and clinical trials, is essential to comprehensively evaluate and confirm the therapeutic potential of THC and CBD in the treatment of cervical cancer.

Cannabinoids, particularly THC, have been shown to inhibit Janus kinase/signal transducer and activator of transcription (JAK/STAT) signalling in T cells (Maia et al. 2023). This effect is mediated through the CB2 receptor, which is expressed on immune cells. CB2 directly interacts with JAK1, suppressing its downstream signalling and leading to reduced phosphorylation of STAT1 and STAT3 (Xiong et al. 2022). By inhibiting the JAK/STAT pathway in T cells, cannabinoids suppress antitumour immune responses (Maia et al. 2023).This highlights the need for caution when using cannabinoid-based therapies in conjunction with immunotherapy, as CB2 receptor activation may interfere with immune-mediated tumour clearance (Xiong et al. 2022). The resulting inhibition of T-cell proliferation and function, including decreased production of IFN-γ and TNF-⍺, can reduce the effectiveness of treatments such as programmed cell death protein 1 (PD-1) blockade (Xiong et al. 2022). Given the intricate relationship between immune mechanisms and tumour biology, ongoing research is focused on strategies to optimise therapeutic outcomes.

The role of cannabinoids in regulating autophagy has received significant interest in cancer research. THC, in particular, has been shown to induce autophagic cell death in cancer cells through several mechanisms. One key pathway involves the induction of endoplasmic reticulum (ER) stress, which triggers the upregulation of stress-responsive proteins such as p8 and TRB3 (Younes et al. 2024). This activation leads to the inhibition of the AKT/mTORC1 signalling pathway, a critical regulator of cellular growth and survival (Salazar et al. 2009). Inhibition of this pathway results in elevated levels of LC3-II, a well-established marker of autophagy, indicating increased autophagic activity (Salazar et al. 2009).

Recent studies have further expanded this understanding by exploring the effects of CBD, both individually and in combination with THC. In breast cancer models, CBD has been found to modulate autophagic pathways by enhancing the expression of LC3B, a protein associated with autophagosome formation (Younes et al. 2024). When combined with THC, a marked synergistic upregulation of LC3B has been observed, suggesting an enhanced autophagic response (Younes et al. 2024).

## Combination therapies: enhancing efficacy with cannabinoids and standard treatments

### Cannabinoids and chemotherapy

The co-administration of cannabinoids with cisplatin has emerged as a promising strategy in oncology (Faiz et al. 2024). This approach is driven by the potential for synergistic interactions that could enhance therapeutic efficacy, especially in overcoming chemoresistance mechanisms associated with cisplatin treatment (Ranasinghe et al. 2022). In vitro and in vivo models have demonstrated that cannabinoids can sensitize tumour cells to cisplatin-induced cytotoxicity, potentially addressing challenges such as efflux pump activation and deoxyribonucleic acid (DNA) repair upregulation (Cherkasova et al. 2023). These findings highlight the potential of cannabinoid-based adjunct therapies to optimize the therapeutic index of cisplatin, suggesting a novel approach for personalized cancer treatment (Cherkasova et al. 2023). While other chemotherapeutic agents like doxorubicin (Table 1) have shown synergistic activity with cannabinoids in treating specific cancers, the focus on cisplatin is relevant due to its widespread use in various cancer types, including cervical cancer (Brown et al. 2019).

**Table 1:** Effects of cannabinoid-chemotherapy combinations on various cancer types in preclinical and clinical studies

Cannabinoid	Chemotherapy drug	Cancer type	Mechanisms/effects	Study type	Reference
CBD	Cisplatin	Head and neck squamous cell cancer	Downregulation of genes involved in DNA replication and repair processes, including *MCM2*, *PARP 1*, and *BRCA1*. Increased expression of p21, which is associated with cell cycle arrest	In vivo	Go et al. (2020)
	Doxorubicin	Triple-negative breast cancer	Inhibits P-glycoprotein mediated drug efflux	In vivo	Surapaneni et al. (2022)
	Gemcitabine	Pancreatic cancer	Inhibition of GPR55 signalling via suppressed MAPK activation, reducing tumour cell growth	In vivo	Ferro et al. (2018)
	Temozolomide	Glioblastoma	Downregulation of MGMT expression, making cells more sensitive to drug	In vivo	Soroceanu et al. (2022)
THC	Temozolomide	Glioblastoma	Enhance autophagy-mediated apoptosis	In vivo	Torres et al. (2011)
	Cytarabine	Leukaemia	Enhance cytotoxic effects through reduced p42/44 MAPK activity	In vitro	Liu et al. (2008)
	Vincristine	Leukaemia	Down-regulation of NF-kB pathway	In vitro	Liu et al. (2008)
THC + CBD	Vinblastine	Leukaemia	Enhance cytotoxic effect of vinblastine in resistant leukaemia cells via down-regulation of P-glycoprotein	In vitro	Hinz and Ramer (2019)
	Carfizilomib	Multiple myeloma	Increased activation of caspase-3 and caspase-6 Decrease in the expression of matrix metalloproteinases (MMPs)	In vitro	Nabissi et al. (2016)
	Temozolomide	Glioma	Improve survival rate (1 year)	Clinical study	Twelves et al. (2021)

Deoxyribonucleic acid (DNA), *m*inichromosome maintenance complex component 2 (MCM2), Poly-adenosine diphosphate-ribose polymerase 1 (PARP1), breast cancer susceptibility gene 1 (BRCA1), G protein-coupled receptor 55 (GPR55), O6-methylguanine-DNA methyltransferase (MGMT)

The PI3K/AKT signalling pathway plays a pivotal role in the progression and metastasis of cervical cancer, making it an appealing target for therapeutic strategies (Rascio et al. 2021; Ye et al. 2024) Targeting this pathway, along with the Bax/Bcl-2/caspase-3 axis involved in apoptosis regulation, presents a compelling approach for cannabinoid-based combination therapies (Rascio et al. 2021). Notably, a study investigating the combination of cannabidiol (CBD) with dasatinib in lung cancer revealed synergistic effects through modulation of both the PI3K/AKT and Bax/Bcl-2/caspase-3 pathways (Ye et al. 2024). While this research was conducted in a lung cancer model, the molecular pathways targeted are also critically involved in cervical cancer. As such, the therapeutic potential of similar combination treatments in cervical cancer appears promising and warrants further investigation.

Clinical studies have shown that combining cannabinoids such as CBD and THC with chemotherapy agents like cisplatin, may mitigate chemotherapy-induced adverse effects such as nausea and vomiting (Bathula and Maciver 2023). This combination has also demonstrated potential in exerting direct antitumour effects in certain malignancies (Bathula and Maciver 2023). However, additional research is needed to clarify the underlying mechanisms and establish optimal treatment protocols for cisplatin-based regimens. A significant clinical limitation lies in the prevailing focus on using cannabinoids primarily for managing chemotherapy-induced side effects (Woerdenbag et al. 2023). This narrow application hinders the broader investigation of their potential as therapeutic adjuncts, particularly in combination with agents like cisplatin, where they may enhance anticancer efficacy beyond symptom relief.

Several cannabinoid metabolites have shown potential in enhancing the effects of chemotherapy, including cisplatin. For instance, THC-COOH's anti-inflammatory properties could potentially reduce cisplatin-induced inflammation, while 11-OH-THC and CBD-7-oic acid may contribute to enhancing the antiproliferative effects of cisplatin (DeLong, et al. 2010). Furthermore, using nanoparticle-based delivery systems to co-deliver cannabinoids and cisplatin could enhance the targeted delivery of both agents to tumour microenvironments, improving selectivity and minimizing off-target effects (Duan et al. 2016). This integrative approach paves the way for precision oncology strategies that capitalize on the synergistic potential of cannabinoids and cisplatin. While these interactions show promise, research focusing on cannabinoids and cisplatin in cervical cancer treatment is currently limited. More studies are needed to fully understand the potential benefits and risks of combining these compounds with cisplatin in cervical cancer treatment and other malignancies where cisplatin is a primary chemotherapeutic agent.

#### Cannabinoids mechanisms for overcoming chemotherapy drug resistance

While direct evidence of cannabinoids specifically inhibiting P-glycoprotein (P-gp) in cervical cancer is scarce, some studies have reported that cannabinoids can modulate drug resistance mechanisms involving efflux transporters like P-gp (Holland et al. 2006). Cancer cells often upregulate these transporters to expel chemotherapeutics and resist treatment (Pilotto Heming et al. 2022). By inhibiting these pumps, cannabinoids can enhance intracellular drug accumulation and cytotoxicity, aiding in overcoming resistance (Pilotto Heming et al. 2022). Cannabinoids have been shown to induce the generation of ROS within cancer cells, resulting in oxidative stress (Pagano et al. 2021). This stress damages essential cellular components, including lipids, proteins, and DNA, thereby activating cell death pathways such as apoptosis and autophagy (Pagano et al. 2021). By promoting ROS accumulation, cannabinoids can sensitize therapy-resistant cancer cells, effectively overcoming resistance mechanisms that rely on robust antioxidant defences (Zhou et al. 2023).This insight into the role of ROS in cancer therapy supports further investigation into modulatory agents like cannabinoids.

In addition to ROS generation, cannabinoids also stimulate autophagy, a cellular degradation process that, when overactivated, can lead to cell death (Younes et al. 2024). In cannabinoid-treated cancer cells, autophagy has been observed to precede apoptosis, suggesting that its initiation sets the stage for subsequent programmed cell death. This sequence enhances the susceptibility of resistant cancer cells to cannabinoid-induced cytotoxicity (Velasco et al. 2016) underscoring their potential as powerful therapeutic agents in overcoming cancer treatment resistance.

Additionally, cannabinoids inhibit the AKT signalling pathway, which supports cell survival, proliferation, and drug resistance (Velasco et al. 2016). Inhibiting AKT leads to cell cycle arrest, typically at the G1/S checkpoint, downregulation of pro-survival proteins, and activation of apoptotic proteins, thereby reducing cancer cell proliferation and enhancing treatment sensitivity (Nitulescu et al. 2018). Emerging studies indicate that cannabinoids may interact with genes involved in therapeutic resistance. For example, the growth factor midkine (MDK), which is linked to resistance to various treatments, promotes this effect by activating anaplastic lymphoma kinase (ALK) (Saikia et al. 2023). ALK activation has been shown to suppress cannabinoid-induced autophagy and apoptosis, thereby limiting their anticancer potential (Lorente et al. 2011). Targeting these resistance-associated pathways could restore cancer cell sensitivity to cannabinoid therapy, enhance the effectiveness of conventional treatments, and offer a strategy to overcome resistance mechanisms in cancer.

### Cannabinoids and radiotherapy

Cannabinoids are being explored for their potential to modulate the effects of radiotherapy, exhibiting both radiosensitizing and radioprotective properties that could enhance their therapeutic synergy with radiation treatments (Yasmin-Karim et al. 2018). On a molecular level, cannabinoids such as THC and CBD exert anti-inflammatory and antioxidant effects by influencing key signalling pathways, including nuclear factor kappa-light-chain-enhancer of activated B cells (NF-κB), MAPK, and JAK/STAT (Yasmin-Karim et al. 2018). Through this modulation, cannabinoids may help shield normal tissues from radiation-induced DNA damage, apoptosis, and inflammation, potentially improving the therapeutic index of radiotherapy (Yasmin-Karim et al. 2018). Conversely, cannabinoids can enhance radiotherapy effects on tumour cells by activating CB1 and CB2, and modulating pathways such as PI3K/AKT, mTOR, and ROS production, inducing cell cycle arrest, mitochondrial dysfunction, and intrinsic apoptosis (Hosami et al. 2021). They may also sensitize tumour cells to radiation by inhibiting DNA repair pathways and upregulating pro-apoptotic proteins (Hosami et al. 2021). Compelling evidence suggests that CBD can enhance the therapeutic efficacy of radiation therapy when used in combination (Yasmin-Karim et al. 2018). In vitro studies demonstrated significantly increased tumour cell death with the combined use of CBD and radiation, compared to either treatment alone (Yasmin-Karim et al. 2018). These effects were even more pronounced in animal models, where mice receiving the combination therapy showed the greatest reduction in tumour growth and the longest survival times among all treatment groups (Yasmin-Karim et al. 2018). These synergistic outcomes were observed in both lung and pancreatic cancer models, suggesting potential applicability across a range of tumour types.

Additionally, CBD may counteract tumour hypoxia, a major contributor to radiotherapy resistance, by reducing the hypoxic environment within cancer cells (Alfonzetti et al. 2020). Addressing hypoxia is critical, as it significantly impairs the effectiveness of radiation therapy. By mitigating this resistance mechanism, CBD may further enhance the therapeutic potential of radiotherapy.

For example, combined cannabinoid treatment prior to irradiation increased markers of autophagy and apoptosis, leading to greater cell death and tumour volume in preclinical glioma models (Skorzewska and Geca 2024). This mechanism likely applies broadly to cancer cells by priming them to respond better to ionizing radiation (Skorzewska and Geca 2024). Cannabinoids inhibit the AKT signalling pathway, which is often overactive in tumours and contributes to resistance to therapies including radiation (Faiz et al. 2024). By downregulating AKT, cannabinoids promote cell cycle arrest and apoptosis, thereby enhancing radiosensitivity (Faiz et al. 2024). This disruption of survival signalling also helps overcome hypoxia-induced resistance mechanisms. The ER stress response induced by cannabinoids activates pathways (e.g., p8–TRIB3) that lead to autophagy and subsequent apoptosis, sensitizing tumour cells to therapeutic interventions like radiation (Hinz and Ramer 2022). Autophagy induction serves as an upstream trigger for apoptotic cell death, creating a cellular environment less able to survive radiation damage (Hinz and Ramer 2022). Cannabinoids can elevate intracellular ROS levels, causing oxidative stress that damages tumour cells and enhances the effects of radiation, which generates ROS as part of its cytotoxic action (Haghmorad et al. 2025). This amplification of oxidative stress can mitigate hypoxia-driven resistance and support (Mendoza et al. 2025). Cannabinoids such as THC and CBD have demonstrated the ability to increase cancer cell sensitivity to radiation by inducing autophagy and apoptosis (Skorzewska and Geca 2024). In preclinical glioma models, cannabinoid treatment administered prior to irradiation elevated markers of autophagy and apoptosis, resulting in enhanced tumour cell death and significantly reduced tumour volume (Skorzewska and Geca 2024). This priming effect suggests a broader applicability across various cancer types, improving cellular responsiveness to ionizing radiation.

The inhibition of the AKT signalling pathway, a pathway frequently overactivated in tumours and associated with resistance to therapies, including radiation, is one of the key mechanisms underlying this radiosensitising effect (Faiz et al. 2024). Cannabinoid-induced downregulation of AKT promotes cell cycle arrest and apoptosis, thereby enhancing radiosensitivity and disrupting survival signals that often support tumour resistance, particularly under hypoxic conditions. Additionally, cannabinoids activate the ER stress response, particularly through the p8–TRIB3 axis, leading to autophagy and subsequent apoptotic cell death (Hinz and Ramer 2022). In this context, autophagy acts as an upstream trigger that compromises the cell's ability to withstand radiation-induced damage. Furthermore, cannabinoids elevate intracellular levels of ROS, intensifying oxidative stress within tumour cells. Since radiation therapy also relies on ROS generation for its cytotoxic effects, this cannabinoid-induced amplification of oxidative stress enhances therapeutic efficacy (Haghmorad et al. 2025). Importantly, this mechanism also helps overcome hypoxia-induced resistance, a major barrier to effective radiotherapy (Mendoza et al. 2025).

Together, these synergistic actions, modulating survival pathways, inducing ER stress, triggering autophagy, and increasing ROS, highlight the potential of cannabinoids to serve as effective radiosensitizers. Their ability to target multiple resistance mechanisms simultaneously offers a promising strategy to improve cancer treatment outcomes when combined with radiotherapy (Hu et al. 2011; Yao et al. 2020). To overcome current challenges and deepen our understanding, several promising research avenues warrant exploration. Firstly, omics-based approaches, such as genomics, proteomics, and metabolomics, could help identify key molecular biomarkers and signalling pathways involved in cannabinoid-mediated radiosensitization and radioprotection. These insights may uncover targets for more precise therapeutic interventions.

In parallel, the development of nanoparticle-based delivery systems for cannabinoids offers a strategy to improve spatial and temporal control of drug release within the tumour microenvironment. This approach could enhance treatment selectivity, reduce off-target effects, and support the design of personalised cervical cancer therapies that fully leverage the synergistic potential of radiotherapy and cannabinoids.

### Cannabinoids in immunotherapy and targeted therapy

**Fig. 2:**
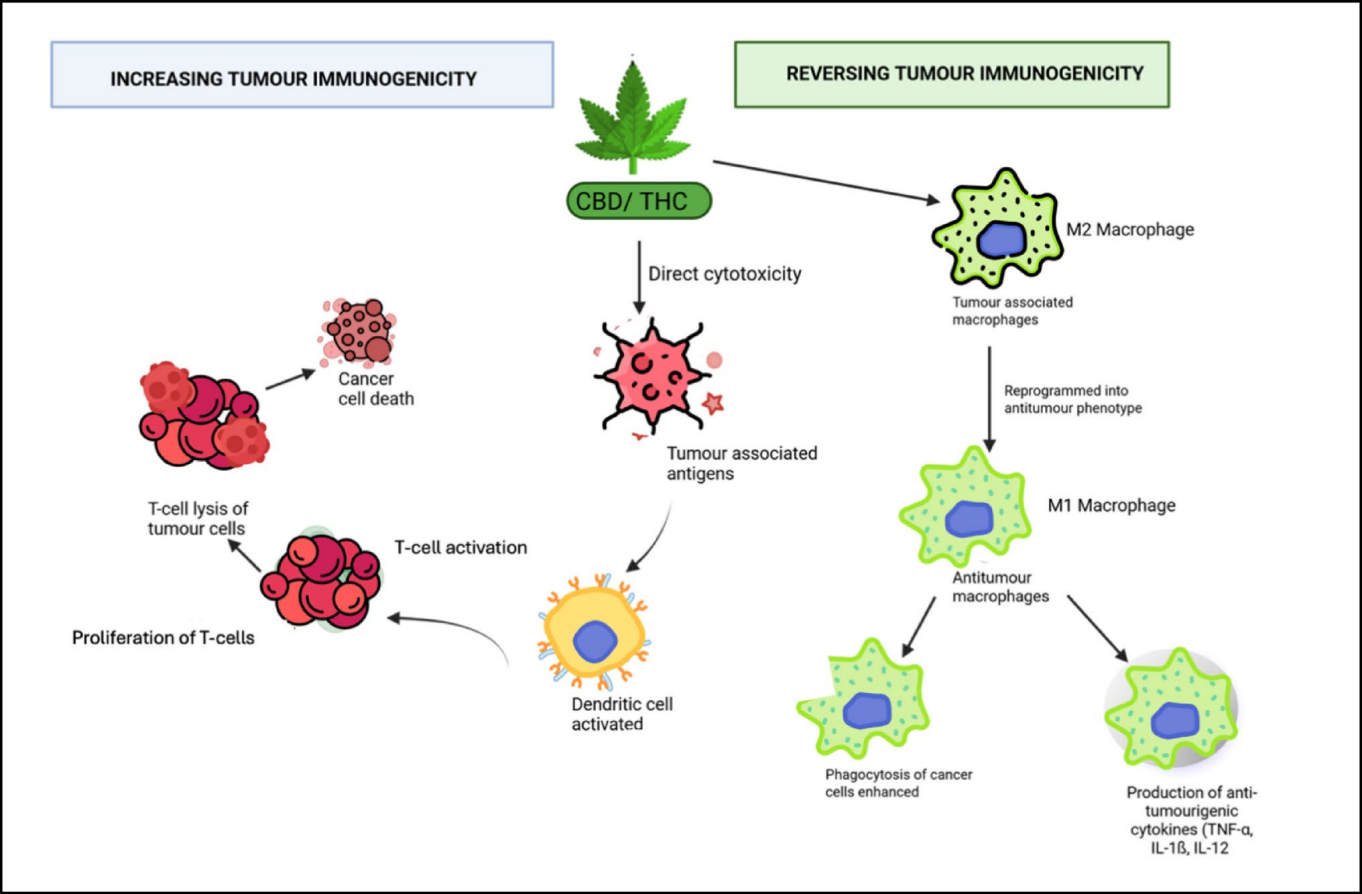
Mechanisms in which cannabinoids may enhance cervical cancer immunotherapy. Figure created by SP Mathibela using FlatIcon.com and PowerPoint Presentation 2021

Cannabinoids have shown promising potential in enhancing cancer immunotherapy by increasing tumour immunogenicity and counteracting tumour-induced immunosuppression (Zaiachuk et al. 2021). Compounds such as CBD and THC exert cytotoxic effects on tumour cells, leading to the release of tumour-associated antigens (Fig. 2) (Zaiachuk et al. 2021), thereby improving tumour recognition by the immune system. This antigen release enhances activation of antigen-presenting cells and promotes a more robust T-cell-mediated immune response (Zaiachuk et al. 2021).

In triple-negative breast cancer (TNBC), CBD has been found to activate the cyclic GMP–AMP synthase–stimulator of interferon genes (cGAS–STING) pathway, resulting in upregulation of PD-L1 expression (Kim et al. 2024). Although increased PD-L1 can dampen T-cell activity, combining CBD with immune checkpoint inhibitors can reverse this suppression (Zaiachuk et al. 2021). This combination enhances cytokine production and strengthens T-cell-mediated tumour cytotoxicity (Fig. 2). These effects have been validated in vivo, where combination therapy resulted in significant tumour growth inhibition, increased infiltration of CD8 + T cells, and elevated granzyme B expression, all indicative of enhanced antitumour immunity (Kim et al. 2024).

Cannabinoids have been shown to reverse the immunosuppressive tumour microenvironment (Zaiachuk et al. 2021). Activation of the CB2 receptor on immune cells enhances their cytotoxic activity and cytokine production, thereby improving immune surveillance. Additionally, cannabinoids can reprogram tumour-associated macrophages from the M2 pro-tumourigenic phenotype to the M1 antitumourigenic phenotype (Zaiachuk et al. 2021). M1 macrophages secrete immunostimulatory cytokines and promote the phagocytosis of cancer cells, contributing to a more robust antitumour immune response (Zaiachuk et al. 2021).

Cannabinoids also modulate immune checkpoints, particularly the PD-1/PD-L1 axis, restoring T-cell function and enhancing the effectiveness of immune checkpoint inhibitors (Kim et al. 2024). These immunomodulatory effects have been demonstrated in TNBC and colorectal cancer models, suggesting potential applications in cervical cancer therapy. Given their capacity to reshape tumour immunogenicity and the immune landscape, cannabinoids represent promising adjuncts to immunotherapy.

Future research should prioritise identifying cannabinoid-responsive pathways and developing nanoparticle-based delivery systems to enhance drug selectivity, tumour accumulation, and overall therapeutic efficacy. Integrating cannabinoids into immunotherapeutic strategies could significantly improve treatment outcomes across various cancer types.

### Challenges in cannabinoid therapeutics

Cannabinoid therapeutics face several challenges, with bioavailability and pharmacokinetics being the key factors. The limited bioavailability of cannabinoids due to poor water solubility necessitates advanced delivery methods to enhance absorption and ensure therapeutic efficacy (Muta et al. 2025). The complex pharmacokinetics of cannabinoids, influenced by liver metabolism, lead to variability in effects based on factors such as dose, administration route, and individual metabolism (Grotenhermen 2003). Legal and regulatory considerations further complicate the development and distribution of cannabinoid-based treatments (Adebisi and Quazeem Olaoye 2022). Strict regulations governing cannabis cultivation, production, and use in many regions create barriers to research and limit access to potential therapies (Adebisi and Quazeem Olaoye 2022). Moreover, regulatory frameworks often lag behind scientific advancements, making it challenging for pharmaceutical companies to efficiently and safely bring cannabinoid-based treatments to market (Hossain and Chae 2024). In vivo studies using animal models have further validated the efficacy of cannabinoids in reducing tumour burden and increasing treatment response (Hinz and Ramer 2022). The psychoactive components of cannabinoids, especially THC, poses significant challenges to their widespread adoption in medical treatments (Arnold 2021). These psychoactive effects can include altered perception, mood changes, impaired cognition and potential addiction, which may be undesirable for patients and healthcare providers (Arnold 2021). The presence of psychoactive components raises concerns about patient safety, drug interactions, dosage control, long term effects and variability in patient responses (Arnold 2021). To address these challenges, research efforts are focusing on developing cannabinoid formulations with reduced psychoactive effects (Lafaye et al. 2017). While progress is being made in this area, there are still significant gaps in clinical research. Clinical studies on cannabinoid-based treatments for cervical cancer remain limited, with most clinical trials focusing on palliative effects rather than direct anticancer effects (Velasco et al. 2016). Challenges such as variability in cannabinoid formulations, lack of standardized dosing protocols, and regulatory restrictions hinder the progression of cannabinoid-based therapies to mainstream clinical applications (Faiz et al. 2024). To establish the safety, efficacy, and therapeutic potential of cannabinoids in cervical cancer treatment, additional well-designed clinical trials are essential.

## Nanotechnology in cervical cancer drug delivery

### Nanocarriers for enhanced cannabinoid delivery in cervical cancer treatment.

Nanocarriers are minuscule systems designed to enhance the efficacy of drugs Various types of nanocarriers exist, each offering distinct advantages. Lipid-based nanocarriers, composed of fats like phospholipids, triglycerides, or cholesterol, include liposomes, solid lipid nanoparticles (SLNs), and nanostructured lipid carriers (NLCs) (Assadpour et al. 2023). These carriers can encapsulate both hydrophilic and hydrophobic drugs, enhancing their solubility, stability, and bioavailability (Himiniuc et al. 2022). SLNs can reduce the required drug dosage, while NLCs safeguard sensitive ingredients (Himiniuc et al. 2022). Polymeric nanocarriers, made from biocompatible materials like poly (lactic-co-glycolic acid) or chitosan, naturally degrade and provide sustained drug release (Himiniuc et al. 2022).They can also transport entities like antibodies, DNA, or RNA to specific targets with minimal adverse effects from degradation products (Himiniuc et al. 2022). Dendrimeric nanocarriers resemble tiny trees with numerous branches, capable of holding substantial drug quantities and releasing them in a controlled manner (Himiniuc et al. 2022). They can also present antigens on their surfaces (Himiniuc et al. 2022). Carbon-based nanocarriers, such as single-walled and multi-walled carbon nanotubes, possess unique thermal, electrical, and mechanical properties, making them valuable for imaging, targeted drug delivery, and tumour ablation (Himiniuc et al. 2022).

Metallic nanocarriers, including gold, silver, and iron oxide nanoparticles, exhibit unique surface properties that enhance the anticancer efficacy of drugs (Himiniuc et al. 2022). Silver nanoparticles also possess anti-inflammatory and antibacterial properties, while iron oxide nanoparticles are effective for imaging, therapy, and drug delivery (Himiniuc et al. 2022). Inorganic nanocarriers like mesoporous silica and selenium nanoparticles can accommodate large drug loads (Himiniuc et al. 2022). Mesoporous silica is versatile, and selenium exhibits anticancer properties (Himiniuc et al. 2022). Micellar nanocarriers, composed of specialized block copolymers, excel at solubilizing hydrophobic drugs and prolonging their circulation in the bloodstream, thereby enhancing their therapeutic effect (Himiniuc et al. 2022).

Although limited research directly addresses cannabinoid delivery or combination therapies for cervical cancer, the attributes of various nanocarriers suggest promising strategies for improving cannabinoid delivery and developing combination therapies. Lipid-based nanocarriers, such as liposomes and nanostructured lipid carriers, could effectively encapsulate hydrophobic cannabinoids, enhancing their solubility and bioavailability (Saengkrit et al. 2014). Polymeric nanoparticles offer controlled release capabilities to sustain therapeutic cannabinoid levels over time (Himiniuc et al. 2022). For combination approaches, nanocarriers like gold nanoparticles have demonstrated enhanced anticancer activity when conjugated with drugs, indicating potential for cannabinoid-chemotherapy combinations (Sinha et al. 2006). Functionalized nanocarriers, such as antibody-decorated dendrimers, could target cannabinoids to specific receptors or cancer cells (Himiniuc et al. 2022). Micellar nanocarriers may increase the systemic bioavailability of cannabinoids (Himiniuc et al. 2022). Innovative methods like photothermal treatment with carbon nanotubes could be explored for dual-action therapy with cannabinoids (Himiniuc et al. 2022). The penetration capacity of polymeric nanoparticles may facilitate more effective delivery of cannabinoids to tumour sites (Himiniuc et al. 2022). While specific research on nanocarrier-based cannabinoid delivery in cervical cancer is necessary, these nanocarrier properties suggest several promising strategies for enhancing cannabinoid delivery and developing combination therapies. The optimal nanocarrier choice would depend on the specific cannabinoid, desired release profile, targeting requirements, and potential combination with other therapeutic agents.

### Role of nanoparticles in drug delivery

Nanoparticle-based drug delivery systems represent an innovative approach to cervical cancer treatment, particularly through the precise targeting of tumour cells (Yao et al. 2020). The incorporation of cannabinoids into nanocarriers has attracted considerable attention due to their structural flexibility, ability to modify surfaces, and capacity to interact with specific cellular receptors (Assadpour et al. 2023). Encapsulating cannabinoids in these nanocarriers improves their physicochemical stability, protects them from early enzymatic breakdown in the bloodstream, and allows for targeted drug delivery with minimal unintended effects (Assadpour et al. 2023).

**Fig. 3:**
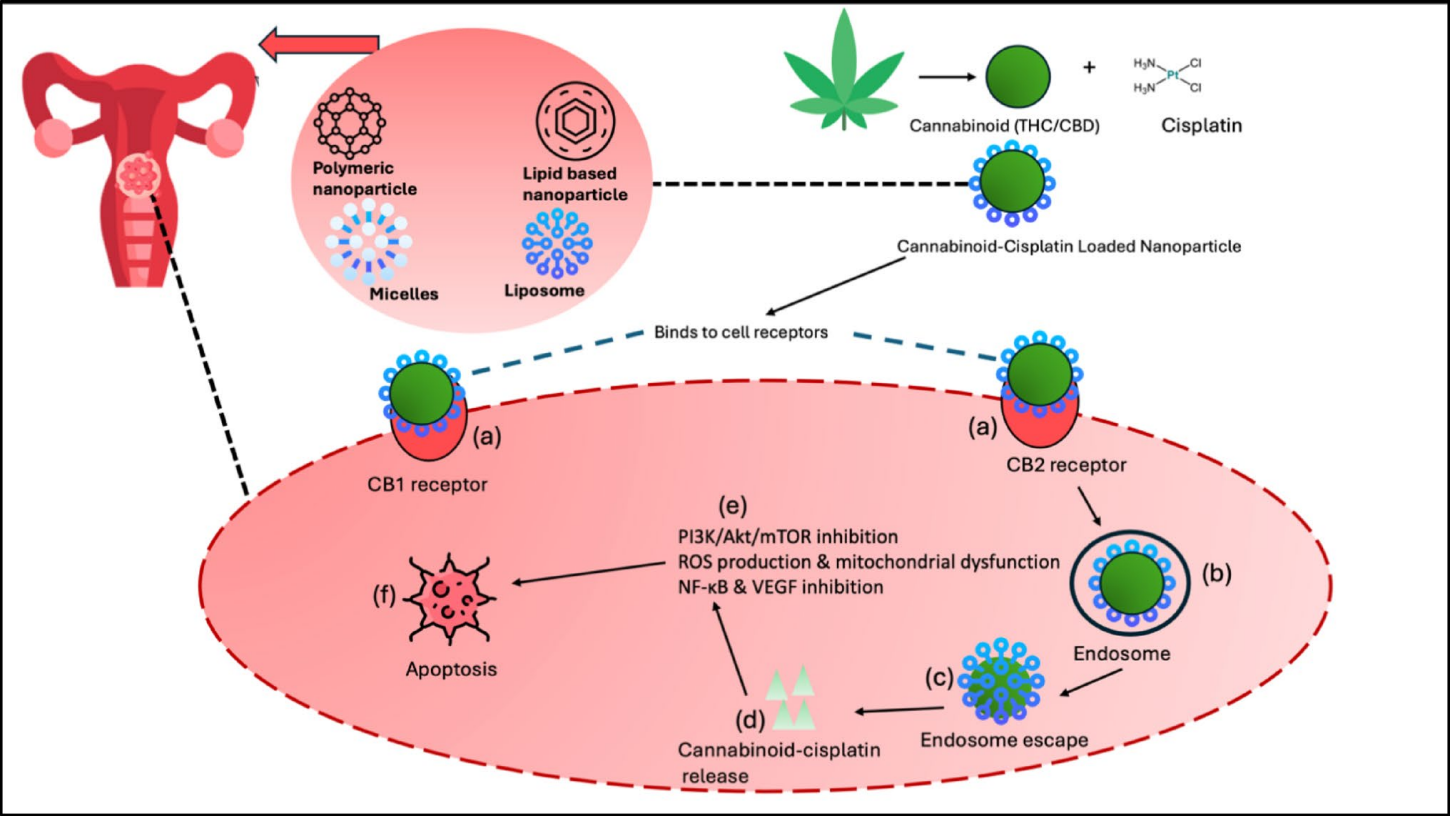
Mechanism of cannabinoid-cisplatin loaded nanoparticle (CCNP) delivery in cervical cancer cells. The figure depicts key stages including targeted delivery, cellular internalisation, endosomal escape, cytoplasmic release, and pathway interactions. The image provides a comprehensive overview of how CCNPs navigate cellular barriers to deliver their therapeutic payload effectively. Figure created by SP Mathibela using FlatIcon.com and PowerPoint Presentation 2021

In the systemic circulation, cannabinoid-cisplatin loaded nanoparticles (CCNPs) exploit the enhanced permeability and retention (EPR) effect to traverse the tumour microenvironment, promoting accumulation at the tumour site (Ordikhani et al. 2016). For localized cervical cancer therapy, however, direct intravaginal or intratumoral delivery of nanoparticles ensures better drug bioavailability by avoiding systemic metabolism and minimizing exposure to healthy tissues (Ordikhani et al. 2016). To achieve targeted delivery to tumours, CCNPs are modified with ligands like folate or transferrin, which bind to receptors that are overexpressed in cervical cancer cells (Fig. 3a) (Ordikhani et al. 2016). Once the ligand-receptor interaction occurs, the CCNPs are internalized through receptor-mediated endocytosis via clathrin-dependent or caveolae-mediated pathways, leading to the formation of intracellular early endosomes (Fig. 3b) (Ordikhani et al. 2016). These endosomes then mature into late endosomes and merge with lysosomes, where unmodified nanoparticles face the risk of enzymatic degradation (Patel et al. 2019). To prevent this, pH-responsive or surface-engineered nanoparticles use escape strategies such as the "proton sponge effect" in polymeric nanoparticles, which causes osmotic swelling and membrane rupture, or lipid-based fusion with the endosomal membrane (Fig. 3c), ensuring the release of cannabinoids into the cytosol (Shinn et al. 2022).

Once released into the cytoplasm (Fig. 3d), cannabinoids and cisplatin interact with several cancer-related signalling pathways. They interfere with the PI3K/AKT/mTOR pathway, thereby hindering tumour growth and survival processes (Fig. 3e) (Lee et al. 2021). Additionally, by inhibiting the NF-κB pathway, they reduce inflammatory and pro-survival signals, while blocking vascular endothelial growth factor (VEGF) signalling to prevent angiogenesis, effectively depriving the tumour of vital nutrients (Pagano et al. 2021).

Another novel strategy to enhance therapeutic effectiveness involves co-delivering cannabinoids with RNA-based treatments, such as small interfering RNA (siRNA) or microRNA (miRNA), encapsulated in nanocarriers (Zare et al. 2022). These RNA molecules can specifically target and silence oncogenes or restore tumour-suppressor functions, creating a synergistic effect with cannabinoids (Kesharwani et al. 2012). Moreover, hybrid nanocarriers that include magnetic or photothermal-responsive components offer a dual-action method, allowing for targeted drug activation through external stimuli like heat or magnetic fields, further enhancing tumour eradication while reducing systemic toxicity (Graham et al. 2025). This innovative combination of nanotechnology and cannabinoid-based therapy highlights the potential of precision medicine in cervical cancer, providing new opportunities for improved drug effectiveness, fewer side effects, and better patient outcomes.

## Future perspective and clinical translation

### Overcoming current limitations

Although cannabinoids hold significant promise in cancer therapy, several challenges must be overcome to enable their successful clinical application. One major limitation is the poor bioavailability and therapeutic efficacy of cannabinoids, which can be addressed through improved formulation and delivery systems (Palrasu et al. 2022). Nanocarriers, such as lipid-based and polymeric nanoparticles, represent a promising strategy by encapsulating cannabinoids and enabling targeted delivery to tumour cells (Himiniuc et al. 2022). These carriers enhance the solubility, stability, and controlled release of cannabinoids, thereby boosting their therapeutic potential while reducing adverse effects (Himiniuc et al. 2022).

Another significant obstacle is the lack of standardization in cannabinoid-based treatments, including variations in dosage, formulation, and routes of administration (Jugl et al. 2021). Addressing these issues requires well-designed clinical trials to determine optimal therapeutic protocols. Additionally, the development of standardized methods for cannabinoid therapy is essential to ensure consistent clinical outcomes and to equip healthcare providers with reliable treatment guidelines.

### Potential for personalised medicine

The future of cannabinoid-based therapies in cancer treatment is closely tied to their integration into personalized medicine, where treatment decisions are guided by patient-specific factors. This approach relies on identifying biomarkers that can predict how cancer cells will respond to various treatment modalities, including cannabinoids, chemotherapy, and radiotherapy (Das et al. 2023). By analysing the genetic and molecular characteristics of an individual's tumour, it may become possible to tailor cannabinoid treatment to maximize efficacy and minimize adverse effects (Babayeva and Loewy 2023). The use of biomarkers could help identify patients who are more likely to benefit from cannabinoid-based therapies or those who may require combination therapies with conventional treatments (Babayeva and Loewy 2023). This personalized approach has the potential to lead to more targeted and effective treatments for cervical cancer, ultimately improving patient outcomes and reducing the trial-and-error approach often seen in cancer therapy.

### Regulatory and ethical considerations

The integration of cannabinoids into standard clinical practice faces significant regulatory and ethical challenges (Adebisi and Quazeem Olaoye 2022). The legal status of cannabis and cannabinoid-based products varies widely across the globe, with some countries adopting strict regulations while others have approved cannabinoids for medical use (Abuhasira et al. 2018). Even in countries where medical cannabis is legal, the regulatory framework for cannabinoid-based therapies is still evolving, and numerous barriers exist in ensuring the quality, safety, and efficacy of these treatments (Hossain and Chae 2024). Regulatory bodies such as the United States Food and Drug Administration and the European Medicines Agency have begun approving certain cannabinoid formulations for medical use, but more research and clinical evidence are needed to support their widespread use in cancer treatment (Simei et al. 2024). Ethical considerations also arise, particularly regarding patient consent and the potential for misuse or overuse of cannabinoid-based treatments (Glickman and Sisti 2020). Additionally, healthcare providers require clear guidelines on integrating cannabinoids into established treatment protocols while ensuring patient safety. Addressing these regulatory and ethical issues will be crucial for the successful clinical application of cannabinoid-based therapies in cancer treatment.

## Conclusion

The convergence of cannabinoids, nanotechnology, and combination therapies presents a promising frontier in cervical cancer treatment. This approach leverages the synergistic potential of cannabinoids with conventional treatments such as chemotherapy, radiotherapy, and immunotherapy, while using nanotechnology for targeted delivery. The integration of these elements could enhance treatment efficacy and minimize side effects. Nanoparticles play an important role in improving the bioavailability and stability of cannabinoids, facilitating their effective delivery to tumour sites. Simultaneously, cannabinoids may sensitize cancer cells to standard treatments, potentially amplifying their therapeutic impact. However, the successful clinical implementation of these therapies necessitates extensive research and clinical trials to optimize formulations, establish dosing protocols, and ensure safety. Furthermore, clinical studies are essential to explore the possibilities of personalized medicine using biomarkers, enabling tailored treatment approaches for individual patients. As this field advances, ongoing efforts to standardize cannabinoid-based therapies and address regulatory and ethical challenges will be vital in establishing these innovative treatment strategies as mainstream options for cervical cancer patients.

## Data Availability

No datasets were generated or analysed during the current study.
